# Gene Set Enrichment Analysis of Interaction Networks Weighted by Node Centrality

**DOI:** 10.3389/fgene.2021.577623

**Published:** 2021-02-24

**Authors:** Alessandra Zito, Marta Lualdi, Paola Granata, Dario Cocciadiferro, Antonio Novelli, Tiziana Alberio, Rosario Casalone, Mauro Fasano

**Affiliations:** ^1^Department of Science and High Technology, Center of Bioinformatics, University of Insubria, Busto Arsizio, Italy; ^2^Unit of Cytogenetics and Medical Genetics, ASST dei Sette Laghi, Varese, Italy; ^3^Laboratory of Medical Genetics, Ospedale Pediatrico Bambino Gesù, Rome, Italy

**Keywords:** systems medicine, network medicine, gene set enrichment analysis, topological analysis, neurodevelopment, neurodegeneration

## Abstract

Gene set enrichment analysis (GSEA) is a powerful tool to associate a disease phenotype to a group of genes/proteins. GSEA attributes a specific weight to each gene/protein in the input list that depends on a metric of choice, which is usually represented by quantitative expression data. However, expression data are not always available. Here, GSEA based on betweenness centrality of a protein–protein interaction (PPI) network is described and applied to two cases, where an expression metric is missing. First, personalized PPI networks were generated from genes displaying alterations (assessed by array comparative genomic hybridization and whole exome sequencing) in four probands bearing a 16p13.11 microdeletion in common and several other point variants. Patients showed disease phenotypes linked to neurodevelopment. All networks were assembled around a cluster of first interactors of altered genes with high betweenness centrality. All four clusters included genes known to be involved in neurodevelopmental disorders with different centrality. Moreover, the GSEA results pointed out to the evidence of “cell cycle” among enriched pathways. Second, a large interaction network obtained by merging proteomics studies on three neurodegenerative disorders was analyzed from the topological point of view. We observed that most central proteins are often linked to Parkinson’s disease. The selection of these proteins improved the specificity of GSEA, with “Metabolism of amino acids and derivatives” and “Cellular response to stress or external stimuli” as top-ranked enriched pathways. In conclusion, betweenness centrality revealed to be a suitable metric for GSEA. Thus, centrality-based GSEA represents an opportunity for precision medicine and network medicine.

## Introduction

High-throughput data consist in a wide amount of information obtained as the output of last-generation technologies. Nowadays, data from omics approaches are obtained to systematically explore human biology at a cellular or molecular level. This leads to a significant advantage in the study of a biological system in its complexity (Tebani et al., 2016). On the other hand, an analytical method capable of reading, prioritizing, and interpreting such large set of information is needed. The integration of the medical/biological language and the mathematical/computational language in a cross-disciplinary approach represents a challenge (Barabási, 2007). For this reason, new theories and new algorithms have now been generated, and a strong support of bioinformatics tools becomes necessary (Al-Haggar et al., 2013).

Gene set enrichment analysis (GSEA) is a powerful tool for the interpretation of high-throughput expression studies such as mass spectrometry-based proteomics or Next-Generation Sequencing, in order to identify insights into biological processes or pathways underlying a given phenotype. Thanks to the acquisition of an expression profile, the list of differentially expressed genes is ranked in terms of a metric associated to the observed expression change. The ranked gene list is compared to a gene set, i.e., a list of genes known to be associated with a certain biological process, gene ontology (GO), molecular function or pathway. The metric is needed to calculate the enrichment score (ES) that indicates the degree by which a gene set is overrepresented at the extremes of the ranked list (Subramanian et al., 2005).

In the Big Data era, meaningful models and impactful results may only be achieved at the network complexity level. In particular, genomics and quantitative approaches to network-based analysis are combined to advance the frontiers of network medicine (Sonawane et al., 2019). Indeed, the multifactorial approach of network medicine is based on the multiplicity of factors that can alter a system to identify functional connections that link the clinical phenotype to multiple factors (Piñero et al., 2016).

Usually, protein networks are built starting from a list of query proteins and by retrieving the protein–protein interaction (PPI) information from a suitable database. The resulting network may be further enriched by looking for first interactors that might join isolated or distant nodes. The rationale of this approach is that proteins that were identified as phenotype-correlated by chance are likely excluded from the network, whereas proteins that for several reasons were not detected as phenotype-correlated are now reconnected to the network (Fasano et al., 2016). A standard functional analysis of the resulting network usually consists in an over-representation analysis based on the Fisher’s exact test or a GSEA (Monti et al., 2019).

The Graph Theory provides tools to analyze the connectivity structure and the topology of a network. Node centrality and edge betweenness are among the most useful parameters to identify hotspots in a network. The importance of a node is the extent of its involvement in a network, and this can be measured in multiple ways. Common measures include degree, closeness, betweenness and eigenvector centralities (Ashtiani et al., 2018). The latter is at the core of the PageRank and the hypertext-induced topics search (HITS) algorithms for ranking networks (Kleinberg, 1999). The betweenness centrality of a given node takes into account the number of geodesics, or shortest paths, between any couple of nodes that include the considered node over the total number of geodesics that connect any couple of nodes of the graph. When nodes are in distinct components, the geodesic pathway is not defined. Otherwise, the betweenness centrality ranges from 0 to 1. On the other hand, the closeness centrality is inversely proportional to the sum of the shortest paths between the given node and any other node in the network.

Several algorithms have been proposed to combine pathway enrichment analysis with network topological information and their performance has been recently reviewed (Ma et al., 2019). Some of them take into account interconnectivity or adjacency information, whereas other consider centrality measures such as degree or betweenness centrality. Nevertheless, all topology-based pathway enrichment analysis methods require an expression metric that indicate whether a gene is differentially expressed in a group comparison.

Since whole genome sequencing (WGS) and whole exome sequencing (WES) are useful genomics tools to identify genes showing alterations (e.g., single nucleotide variants – SNVs, Indels, larger duplications or deletions), the results generated by these techniques can be used to generate personalized networks for each individual (Suwinski et al., 2019). More generally, altered genes can be considered as constituting the input list for generating a PPI network that reflects a disease network model in a single individual, thus allowing for the comparison between personalized networks of patients with the same phenotype and a distinct genetic background.

Here, we propose a GSEA approach to analyze PPI networks based on betweenness centrality values as ranking metrics. The proposed analysis was applied to two case paradigms. In the first case, the network was obtained from genomics data, where quantitative expression metrics are not available. In the second case, the network represents the result of a secondary data analysis, where proteomics data are scraped from the literature and quantitative information is not always available.

## Materials and Methods

### Definition of Gene Signatures From Genomics Analysis

The patients and their parents came from the cohort of medical genetics clinics of the ASST Sette Laghi showing clinical phenotypes related to neurodevelopmental disorders. A written informed consent to perform array-based comparative genomic hybridization (aCGH) and WES, and a consent for use of anonymous data for research were provided by the parents and relatives of the patients. The consent model and procedure were approved by the Institutional Review Board (ASST Sette Laghi Cod MOD09 IOS01SSDGM). Neurocognitive tests were based on WISC-IV (Wechsler Intelligence Scale for Children). Language skills were assessed with Boston and TROG-2 tests. No bias selection of gender, family history or age was performed. To identify submicroscopic chromosomal rearrangements, the aCGH technology was performed after DNA extraction from peripheral blood cells (QIAmp DNA blood Maxi Kit, Qiagen, Hilden, Germany). The four patients were selected on the basis of the presence of 16p13.11 microdeletion.

For exome results, genes involved in neurodevelopmental disorders and genes related to rare diseases were considered according to literature. Among these, genes with variants in hemizygosity, homozygosity, compound heterozygosity and simple heterozygosity inherited from the healthy non-carrier parent of the 16p13.11 deletion were selected.

Array-based comparative genomic hybridization was performed using CytoSure ISCA V2 4x180K platform with a backbone resolution of 1 probe/25 Kb and 1 probe/19 Kb in critical regions, human genome reference hg19/GRCh37 and sex matched normal human DNA pool (Kreatech, Amsterdam, Holland) as control. InnoScan 710 Microarray Scanner (Carbonne, France) and Mapix (Innopsys, Carbonne, France) were used to detect and analyze fluorescence levels. Results were interpreted using Cytosure Interpret Software (Oxford Gene Technology, Begbroke, Oxfordshire, United Kingdom).

Whole exome sequencing was performed on probands and parents DNA using the Twist Human Core Exome Kit (Twist Bioscience, San Francisco, CA, United States), according to the manufacturer’s protocol and sequenced on the Illumina NovaSeq 6000 platform. The BaseSpace pipeline (Illumina, San Diego, CA, United States) and the TGex software (LifeMap Sciences, Alameda, CA, United States) were used for the variant calling and annotation, respectively. Sequencing data were aligned to the hg19/GRCh37 human reference genome. Variants with a lower coverage than 10×, quality score (GQ) <15, and gnomAD minor allele frequency (MAF) >5% have been excluded.

### Protein–Protein Interaction Networks From Genomics Analysis

Cytoscape 3.7.2^1^ was used to generate networks (Su et al., 2014). The public database IMEx^2^ was queried through Cytoscape using the PSICQUIC standard (the Proteomics Standard Initiative Common QUery InterfaCe). Each list of altered genes, obtained by aCGH and WES as described above, was used to generate a network encompassing all first interactors of the gene products. The network was filtered for taxonomy ID 9606 (Homo sapiens) to remove homology inferences. A first interactors list was generated and used to query the IMEx database again, thus including second interactors too. All self loops and duplicated edges were removed. Node degrees were calculated using the NetworkAnalyzer plugin. Isolated (degree = 0) and terminal (degree = 1) nodes were removed to reduce the network size, with the only exception for isolated/terminal nodes included in the input list of altered genes.

### Protein–Protein Interaction Network From Secondary Data Analysis

Proteins reported to be associated to Parkinson’s disease (PD), amyotrophic lateral sclerosis (ALS) and Alzheimer’s disease (AD) were extracted from a secondary data analysis by Monti et al. (2018). Using a pubmed scraping procedure, authors obtained lists of proteins reported to be quantitatively altered in the three neurodegenerative disorders considered and built a network that encompasses all selected proteins and their common interactors using PPI spider (Antonov et al., 2009). The network was then exported as a.xgmml file.

### Topological Analysis in Cytoscape

Betweenness centrality was calculated for each node in networks by using the NetworkAnalyzer utility in the Cytoscape Environment. Both size and border width of all nodes were mapped onto their betweenness centrality values. The betweenness centrality of node n*_*i*_* is given by $C_{B}\,\left(n_{i}\right)=\sum_{j<K}\frac{\sigma_{j\,k}\,\left(n_{i}\right)}{\sigma_{j\,k}}\,\frac{1}{\left(\begin{array}{c} N\\ 2 \end{array}\right)}$where *N* is the total number of nodes, σ*_*jk*_* is the number of geodesics between nodes *n*_*j*_ and *n*_*k*_, and σ*_*jk*_*(*n*_*i*_) is the number of geodesic pathways containing node *n*_*i*_. Networks were reduced in size by selecting nodes having betweenness centrality ≥10^–4^ for display clarity only. For each network, betweenness centralities were transformed with a Hill function in order to enhance the weight of nodes with higher values, as follows hereafter: $C_{B}^{{}^{\prime }}=\frac{C_{B}^{h}}{k+C_{B}^{h}}$.

### Gene Set Enrichment Analysis Using R

Gene set enrichment analysis was performed using the ReactomePA package (Yu and He, 2016) in the R environment for statistical analysis (R Core Team, 2017). Normalized enrichment score (NES), *p*-value, and false discovery rate (FDR) for all variables and signatures were obtained in the R environment. The database for biological processes/pathways Reactome was used for the analysis (Fabregat et al., 2017). Hill-transformed betweenness centralities were used as a suitable ranked list metric instead of those usually considered in expression studies. The list was then resampled by randomly assigning observed values to gene IDs and the analysis was performed again to identify pathways in common with those arising from the analysis of values that were obtained from the topological analysis of the networks. The R code is reported in the Supplementary Material.

### Over-Representation Analysis

Over-representation analysis was performed for comparison using the WEB-based GEne SeT AnaLysis Toolkit^3^ (Liao et al., 2019) using Reactome as the functional database and “human genome, protein coding” as the reference set.

## Results

### Personalized Signatures From aCGH and WES Analysis

Four patients were referred to genetic investigations for diagnostic purposes and counseling for developmental disorders ranging from learning delay to intellectual disability, with or without associated congenital malformations. By aCGH screening, a microdeletion on 16p13.11 was identified. The deletion on 16p13.11 was either inherited from the healthy mother (two patients) or from the healthy father (one patient), or it was a *de novo* unbalance (one patient). Several mutations inherited from both healthy parents were found in the four patients by WES analysis. By merging aCGH and WES results, lists of altered genes were generated and used as personalized signatures for each patient (Table 1).

**TABLE 1:** Gene signatures for the four patients.^a^

**Patient 1**	**Patient 2**	**Patient 3**	**Patient 4**
*ABCC1*	*ABCC1*	*ABCC1*	*ABCC1*
*ABCC6*	*ABCC6*	*ABCC6*	*ABCC6*
*ANK3*	*BCKDK*	*BCKDK*	*FOPNL*
*DYRK1A*	*CHRNA7*	*FOPNL*	*GPRASP1*
*FOPNL*	*FOPNL*	*IQSEC2*	*MARF1*
*GRIN2A*	*GRM5*	*MARF1*	*MYH11*
*KIF1A*	*MARF1*	*MYH11*	*NDE1*
*MARF1*	*MECP2*	*NDE1*	*NLGN4X*
*MYH11*	*MYH11*	*NOMO3*	*NOMO1*
*NDE1*	*NDE1*	*RGPD4*	*NOMO3*
*NOMO3*	*NOMO3*	*RRN3*	*NPIPA1*
*NPIPA1*	*RRN3*	*TG*	*NRXN1*
*NTAN1*	*SHANK1*	*TSC2*	*NTAN1*
*PDXDC1*	*XYLT1*	*XYLT1*	*PDXDC1*
*RRN3*			*PHEX*
			*RRN3*

*^a^Lists include altered genes from both aCGH and WES results.*

The datasets presented in this study can be found in online repositories. The names of the repository/repositories and accession number(s) are listed hereafter. For aCGH data: DECIPHER^4^ 350680 (Patient 1), 414066 (Patient 2), 414078 (Patient 3) and 318359 (Patient 4). For WES data: European Variation Archive Project: PRJEB41629, Analyses: ERZ1687005.

### Genomics Networks Without Expression Metrics

The four lists of altered genes were used to generate personalized networks. Supplementary Figure 1 shows an overview of the four networks encompassing first and second interactors after filtering out isolated and terminal nodes (i.e., nodes with degree = 0 and degree = 1, respectively) and nodes with betweenness centrality ≤10^–4^, except for genes in the signature lists. Signature genes display different betweenness centrality values in the four personalized models with three orders of magnitude among different genes (Table 2). None of the interaction networks has nodes with high values. Genes *MYH11* and *NDE1* are present in all four networks with relatively high centralities, whereas *ANK3*, *CHRNA7*, *DYRK1A*, *KIF1A*, *SHANK1*, and *TSC2*, although they are present in single networks, also have high centrality. On the other hand, several signature genes have negligible centrality, although they are present in all four networks (e.g., *ABCC1*, *ABCC6*, *FOPNL*, *MARF1*, *NOMO3*, and *RRN3*).

**TABLE 2:** Betweenness centrality values (−log_10_) of all signature genes.

	**Patient 1**	**Patient 2**	**Patient 3**	**Patient 4**
*ABCC1*	3.98	3.87	3.70	3.86
*ABCC6*	5.02	4.80	4.71	4.74
*ANK3*	3.00	–	–	–
*BCKDK*	–	3.11	2.90	–
*CHRNA7*	–	3.18	–	–
*DYRK1A*	2.97	–	–	–
*FOPNL*	4.63	4.69	4.61	4.60
*GPRASP1*	–	–	–	2.97
*GRIN2A*	5.07	–	–	–
*GRM5*	–	5.13	–	–
*IQSEC2*	–	–	4.79	–
*KIF1A*	2.52	–	–	–
*MARF1*	3.87	3.67	3.51	3.58
*MECP2*	–	2.50	–	–
*MYH11*	2.78	2.91	2.74	2.77
*NDE1*	2.53	2.49	2.32	2.38
*NLGN4X*	–	–	–	5.49
*NOMO1*	–	–	–	2.68
*NOMO3*	4.46	3.99	3.85	4.03
*NPIPA1*	0	–	–	0
*NRXN1*	–	–	–	5.55
*NTAN1*	5.27	–	–	4.82
*PDXDC1*	3.03	–	–	2.94
*PHEX*	–	–	–	0
*RGPD4*	–	–	5.49	–
*RRN3*	4.78	4.66	4.51	4.75
*SHANK1*	–	3.45	–	–
*TG*	–	–	0	–
*TSC2*	–	–	2.53	–
*XYLT1*	–	4.19	4.07	–

**FIGURE 1:**
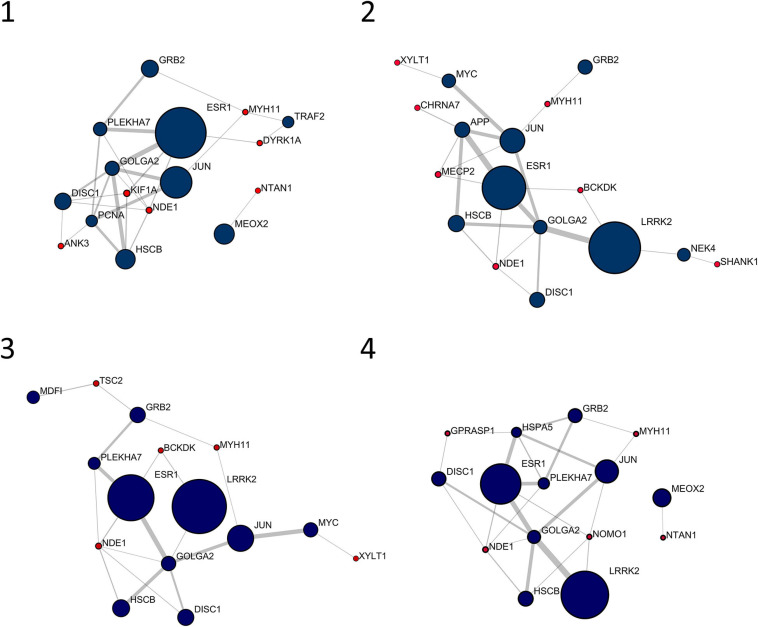
Top 10 nodes in terms of betweenness centrality and interacting signature nodes. Red nodes are genes from the individual gene signature and navy-blue nodes are their first interactors. Diameter and border width of nodes are proportional to betweenness centrality, whereas edge width is proportional to edge betweenness.

Figure 1 shows the 10 most central nodes in each personalized network together with interacting signature genes. In all cases, top 10 nodes are among the first interactors of signature genes, with six out of 10 genes in common among patients (i.e., *DISC1*, *ESR1*, *GOLGA2*, *GRB2*, *JUN*, and *HSCB*). No signature genes displayed centrality values comparable to first interactors represented in Figure 1. An over-representation analysis of these clusters, including top 10 genes and interacting signature genes, did not lead to any significantly over-represented pathway.

**FIGURE 2:**
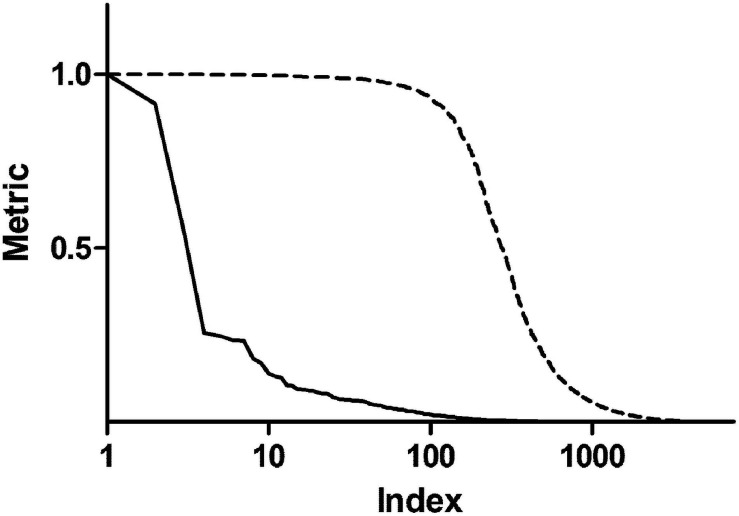
Centrality metrics before and after Hill transformation. The solid line shows betweenness centrality obtained from the topological analysis of a representative personalized network. The dashed line represents centrality values after transformation with a Hill function.

In order to enrich the functional analysis with nodes with higher centrality, original betweenness centrality values were transformed with a Hill function. Figure 2 shows ranked centrality values before and after the transformation. In this way, nodes with low centrality have a negligible contribution to the ES, whereas nodes with high centrality represent leading edges. As an example, patient 1 network had 8,101 nodes. Among them, 579 nodes had a score (i.e., betweenness centrality after Hill transformation) greater than 0.2, 318 greater than 0.5, and 192 greater than 0.8. Therefore, this transformation dramatically reduced the dimensionality of the dataset by selecting less than 10% of the original network genes. As a comparison, we performed the same transformation using closeness centrality. As shown in Supplementary Figure 2, closeness centrality values are distributed in a narrow range even after Hill transformation.

**FIGURE 3:**
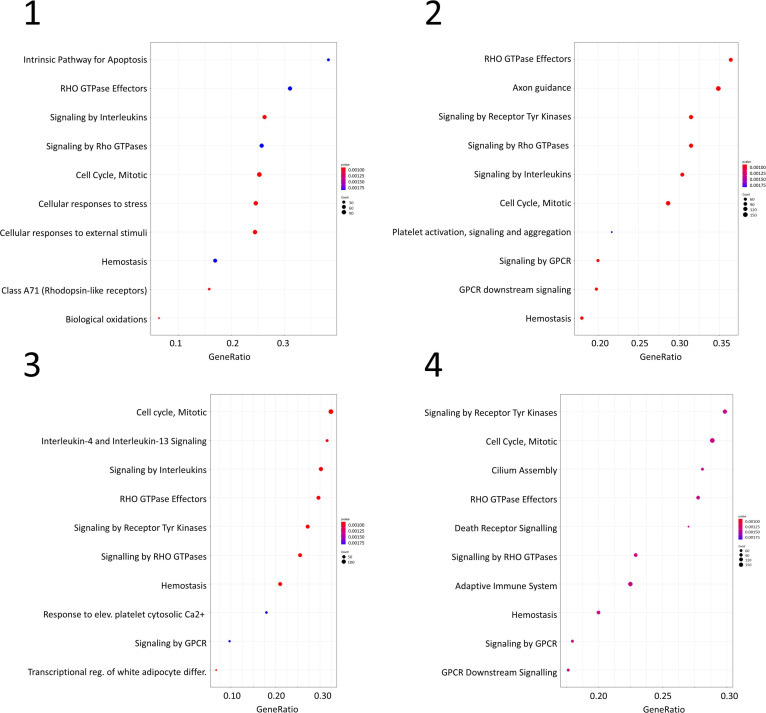
Dot plots for the four GSEA results. Numbers refer to patients as indicated in Table 1. Dot size is proportional to the number of overlapping genes. *p*-values are color-coded according to the color scale.

Gene set enrichment analysis was performed for the four networks using transformed betweenness centrality values as the scoring metric, with Reactome as gene set database. Figure 3 shows enriched categories as a dot plot, with dot size proportional to the number of overlapping genes. All patients displayed the enrichment of one or more pathways related to cell cycle and to Rho GTPases in a different ranking in terms of statistically significant gene ratio, i.e., the ratio between the number of genes related to each pathway and the total number of genes in the network. These pathways were not identified after resampling of scores obtained from betweenness centrality values (Supplementary Figure 3). Moreover, classic over-representation analysis of the whole network without ranking measures led to a different set of enriched pathways. Table 3 summarizes enriched pathways found by classic over-representation analysis of patient 1 network as a whole and by selecting subsets of nodes by their ranking (8,101, 579, 318, and 192 genes, respectively) (see Supplementary Materials).

**TABLE 3:** Comparison of enriched pathways.

**Pathway**	**ORA All^a^ (8101)**	**ORA 0.2 (579)**	**ORA 0.5 (318)**	**ORA 0.8 (192)**	**GSEA**	**GSEA random**
Translation	X					
RNA metabolism	X					X
Deubiquitination	X			X		
Developmental biol.	X					
Organelle biogenesis	X	X				
Vesicle transport	X	X	X			
Transcription	X	X	X	X		
Disease	X	X	X	X		
Immune system	X	X	X	X		X
Cell cycle	X	X	X	X	X	
Apoptosis/Cell death		X	X	X	X	
Cellular resp. to stress/ext. stimuli		X	X	X	X	
Rho GTPases effectors/signaling		X	X	X	X	
Post-transl. modification		X	X			
Cilium assembly			X	X		
Intracellular signaling				X	X	
Hemostasis					X	
Biological oxidations					X	

*^a^ORA All, over-representation analysis performed on all nodes in the network; ORA 0.2, ORA performed on 579 nodes with ranking metric >0.2; ORA 0.5, ORA performed on 318 nodes with ranking metric >0.5; ORA 0.8, ORA performed on 192 nodes with ranking metric >0.8; GSEA, topology-based GSEA using all ranked genes; GSEA random, topology-based GSEA using all ranked genes after resampling.*

### Consensus Networks From Secondary Data Analysis

**FIGURE 4:**
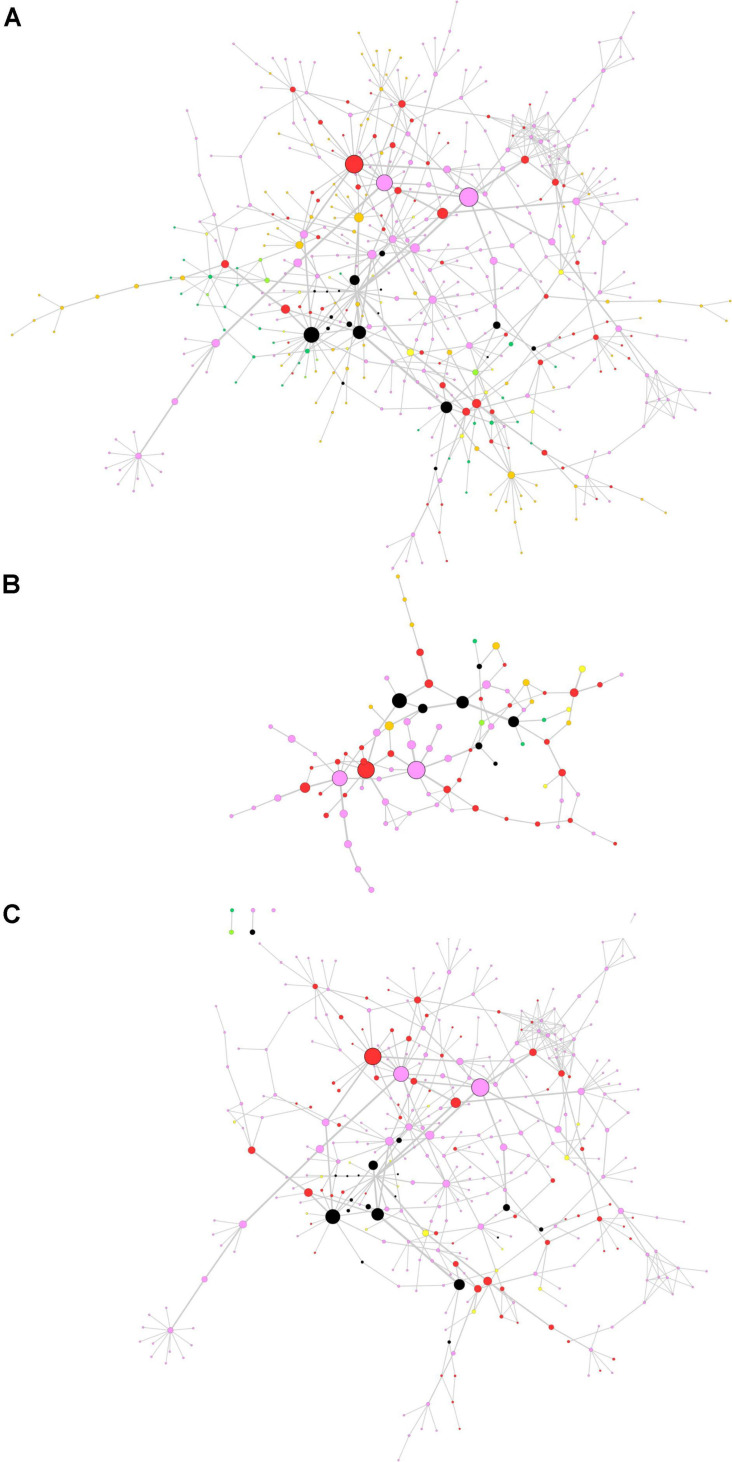
Topological analysis of the proteomics neurodegeneration network from secondary data analysis. **(A)** Whole network; **(B)** top 20% most central nodes; **(C)** selection from the whole network of nodes altered in Parkinson’s disease (PD) patients. Nodes are color-coded as follows. Pink, PD; dark green, amyotrophic lateral sclerosis (ALS); orange, Alzheimer’s disease (AD); yellow, PD and ALS; light green, ALS and AD; red, PD and AD; black, PD, ALS and AD (adapted from Monti et al., 2018). Node size and border width are proportional to betweenness centrality, whereas edge width is proportional to edge betweenness.

The centrality-based GSEA approach was then applied on a consensus network already available in the literature. In a previous paper by Monti et al. (2018), authors retrieved full-text proteomics original articles focused on the development of three neurodegenerative diseases and extracted three input lists including 928 proteins for AD, 1,155 proteins for PD, and 387 proteins for ALS. The input lists were used to generate a consensus network representing interactions among proteins in the lists (Monti et al., 2018). We performed the topological analysis of the consensus network, whose results are shown in Figure 4A, where node size and border width are proportional to betweenness centrality. This network encompasses 483 nodes and 659 edges. Noteworthy, among the 95 nodes with the highest betweenness centrality (i.e., the top 20% central nodes), 78 are linked to PD, either in common or not with other diseases (Figure 4B). Most central genes are *TIMP2* and *EIF3A*, both associated to PD only, and *GABARAPL2*, associated to PD and AD. However, these three genes did not lead to any significant association with PD pathogenesis, when taken alone. All nodes related to PD were then extracted from the whole network for further topological analysis and GSEA (Figure 4C).

**FIGURE 5:**
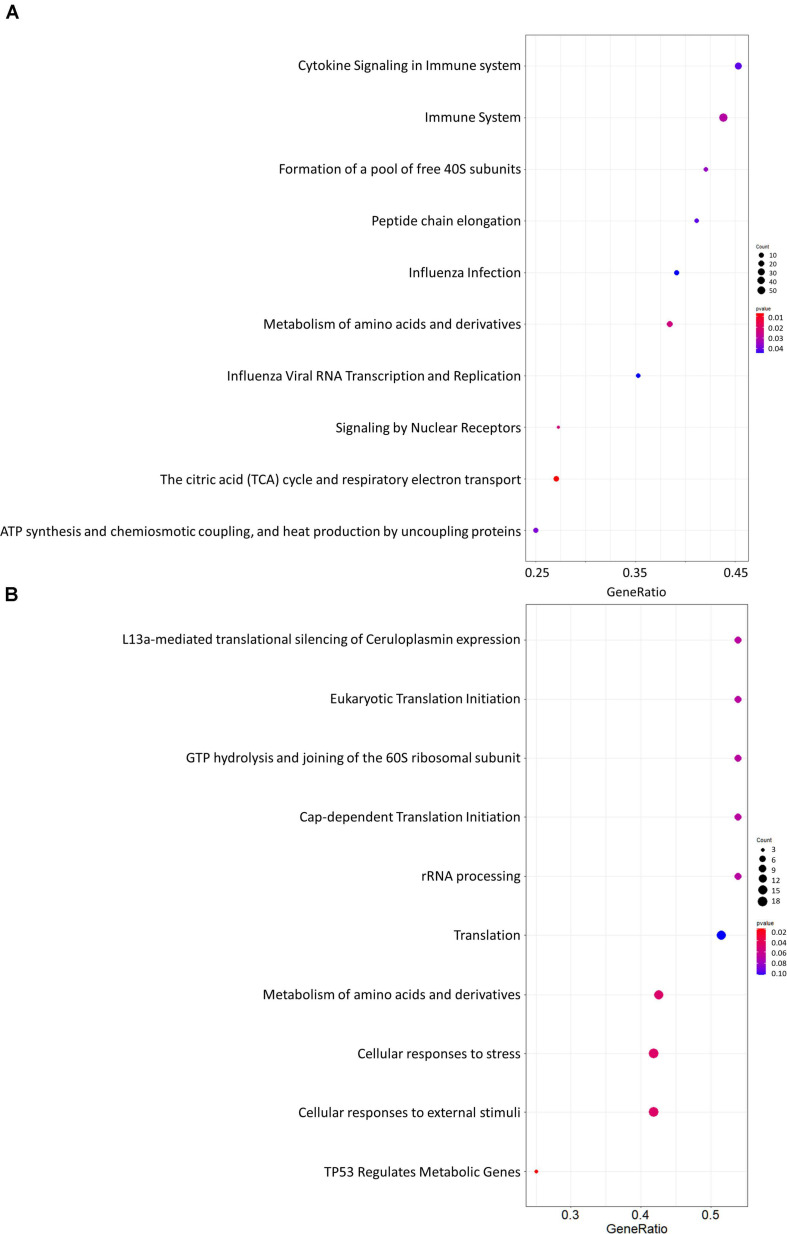
Dot plots for the GSEA results. **(A)** Whole network; **(B)** selection of nodes altered in Parkinson’s disease patients. Dot size is proportional to the number of overlapping genes. *P*-values are color-coded according to the color scale.

Gene set enrichment analysis was performed for the whole network (Figure 4A) and for the PD network (Figure 4C) using Hill-transformed betweenness centrality values as the scoring metrics, with Reactome as gene set reference database. Figure 5 shows enriched categories as a dot plot, with dot size proportional to the number of overlapping genes. For the whole network, enriched pathways mainly refer to catabolic pathways. Immune system appeared to be significantly enriched; however, it was also found after resampling of centrality metrics (Supplementary Figure 4). On the other hand, most significantly enriched pathways for the PD network were “Metabolism of amino acids and derivatives” and “Cellular response to stress or external stimuli,” that were not observed in the analysis of the resampled dataset.

## Discussion

Gene set enrichment analysis is usually performed to associate a disease phenotype to a group of genes by using quantitative expression data. Unlike over-representation analysis, GSEA has the advantage of considering the role of each gene by taking into account its correlation to the clinical phenotype or any other categorical variable (treatment, stage, time, and other experimental conditions) (Subramanian et al., 2005). Nevertheless, a quantitative metric is not always available in a dataset. Here, GSEA based on betweenness centrality of a PPI network is described and applied to two cases, where an expression metric is missing.

Actually, building a network starting from a list of entities such as genes or proteins is based on publicly available evidence of interaction, and the topology of the network accounts for the specific role each protein plays in the network itself. The degree of each node (i.e., the number of interactions with other nodes) is clearly a measure of how much this node is integrated in the network. We decided to consider the betweenness centrality as a suitable metric to rank network nodes in terms of the role they play in the disease represented by the network. Among other centrality measures, betweenness centrality discriminates centrality of nodes over a range that spans orders of magnitudes, unlike closeness centrality values that are distributed in a narrow range and therefore are slightly affected by the resampling procedure. Signature genes of the first case analyzed here showed very different centrality values in a range from 10^–6^ to 10^–3^. Worthy of note, several genes showing high centrality values are involved in neurodevelopmental disorders. Indeed, some of them (*ANK3*, *BCKDK*, *CHRNA7*, *DYRK1A*, *MECP2*, *SHANK1*, and *TSC2*) are included in the database SFARI (Banerjee-Basu and Packer, 2010) with high scores, supporting that the gene contributes to the disorder pathogenesis or it is a strong candidate.

The personalized networks are assembled around a cluster of very central genes, whose composition is partially overlapping. Interestingly, the top 10 clusters never include signature genes, and the weight of every member of the clusters may change among patients. As an example, the leucine-rich repeat kinase 2 (*LRRK2*) is present in all four networks. However, its centrality is low in patient 1 (C*_*B*_* = 7.56 × 10^–3^), whereas it has the top value in the other three patients. Recently, a role for *LRRK2* in cognitive development has been proposed, thus linking *LRRK2* to intellectual disability and autism (Labonne et al., 2020). Another gene present with very high centrality in all four networks, *ESR1*, has been related to the pathogenesis of autism and autism related symptoms (Wang et al., 2016).

We used the tools included in the ReactomePA package (Yu and He, 2016) to associate a topological parameter to each gene in the dataset in place of commonly used expression data. However, we observed that betweenness centrality values show a steep decrease within the first nodes in the ranked list. To select a sub-population of central nodes, betweenness centrality values were transformed with a Hill function that assigns high scores to less than 10% nodes. In this way, a drastic change in the weight associated to each gene is expected. Using Hill-transformed betweenness centrality values, GSEA of the four personalized networks yielded a different ranking of enriched pathways or even different pathways. As an example, the pathway “Cell Cycle, Mitotic” is enriched in all four patient networks. However, its ranking differs in the four dot plots. From the pathogenetic point of view, all patients share a neurodevelopmental impairment that is brought to evidence by the present analysis (e.g., “Mitotic G1-G1/S phase” pathway). It is known from literature that neurodevelopmental disorders are related to alterations of the cell cycle, and a central role is played by genes involved in neurogenesis regulation and cell adhesion processes (Pramparo et al., 2015). Moreover, our evidence supports the conclusions obtained so far on *NDE1*, the candidate gene for the susceptibility to the neurocognitive disorder given by the deletion on 16p13.11. Using a bottom-up approach, *NDE1* and *NDEL1* (its paralogue) were deeply characterized (i.e., biochemistry, PPIs, role in neuronal differentiation and cortical development, pathology caused by their alterations) (Bradshaw and Hayashi, 2017). In particular, the role of *NDE1* and *NDEL1* in cell cycle progression, neurite outgrowth and development and neuronal migration has been highlighted, thus supporting our results.

As a second application of the proposed approach, we analyzed a PPI network previously published by our group (Monti et al., 2018). This network encompasses proteins that were reported to quantitatively change in three neurodegenerative diseases by independent articles. Two of them are movement disorders (PD and ALS) and one is a cognitive disorder (AD). The original aim of the work was to distinguish between proteins that are specifically associated to a single neurodegenerative disease and proteins generically linked to neurodegeneration. Topological analysis of the network adds substance to this purpose. We observed that most central proteins are often linked to PD, either standalone or associated to other disorders. Indeed, the most evident colors in Figure 4B are pink (PD), red (PD and AD) and black (PD, AD and ALS). To gain more evidence to pathways associated to most central nodes, we extracted a subnetwork to represent all proteins that were altered in PD. This selection, together with the topological analysis, allowed us to improve the specificity of GSEA.

The use of a topological parameter to perform GSEA in the absence of any quantitative expression metric was never done before. This application of GSEA has been performed on two datasets, where expression metrics were not available. Although we decided to consider betweenness centrality as a ranking metric, further analyses could be performed by identifying other candidate topological parameters and using these data to obtain new pathway networks and new information.

## Conclusion

The centrality-based GSEA procedure is another powerful tool to investigate personalized networks or disease networks, in order to unveil hidden information and to specifically plan experimental investigations. This analysis can be applied to a wide variety of networks, also in combination with clustering tools. Since biological networks are usually too large and are characterized by high modularity, topological descriptors could assist the identification of functional modules (Sharma et al., 2017; Choobdar et al., 2019). Altogether, the functional analysis of networks by weighting nodes in terms of their centrality could also provide a valuable tool to explore pathogenetic mechanisms and to precisely identify sensitive targets for drug development or repositioning. In this view, centrality-based GSEA represents an opportunity for precision medicine and network medicine.

## Data Availability Statement

The datasets presented in this study can be found in online repositories. The names of the repository/repositories and accession number(s) can be found below: aCGH data: DECIPHER https://decipher.sanger.ac.uk/350680 (Patient 1), 414066 (Patient 2), 414078 (Patient 3) and 318359 (Patient 4). WES data: European Variation Archive Project: PRJEB41629, Analyses: ERZ1687005.

## Ethics Statement

The studies involving human participants were reviewed and approved by ASST Sette Laghi ethical committee. Written informed consent to participate in this study was provided by the participants’ legal guardian/next of kin.

## Author Contributions

AZ performed the cytogenetic analysis, bioinformatics analysis and wrote the manuscript. ML and TA revised the GSEA results and the manuscript. PG performed the cytogenetic analysis and contributed to the interpretation of whole exome sequencing results. DC and AN performed the whole exome sequencing analysis. RC enrolled the patients, carried out medical genetics visits, supervised genetics analysis, and revised the manuscript. MF supervised the project, wrote the code, and wrote the manuscript. All authors approved the final version of the manuscript.

## Conflict of Interest

The authors declare that the research was conducted in the absence of any commercial or financial relationships that could be construed as a potential conflict of interest.

## Footnotes

1 https://cytoscape.org/

2 https://www.imexconsortium.org/

3 www.webgestalt.org

4 https://decipher.sanger.ac.uk/

## Abbreviations

aCGH: array-based comparative genomic hybridization
AD: Alzheimer’s disease
ALS: amyotrophic lateral sclerosis
ES: enrichment score
GO: gene ontology
GSEA: gene set enrichment analysis
HITS: hypertext-induced topics search
MAF: minor allele frequency
NES: normalized enrichment score
PD: Parkinson’s disease
PPI: protein–protein interaction
SNV: single nucleotide variant
WES: whole exome sequencing.

## References

[B1] Al-Haggar M. M. Khair-Allaha B. A. Islam M. M. Mohamed A. S. A. (2013). Bioinformatics in high throughput sequencing: application in evolving genetic diseases. *J. Data Mining Genomics Proteomics* 4:131. 10.4172/2153-0602.1000131

[B2] Antonov A. V. Dietmann S. Rodchenkov I. Mewes H. W. (2009). PPI spider: a tool for the interpretation of proteomics data in the context of protein-protein interaction networks. *Proteomics* 9 2740–2749. 10.1002/pmic.200800612 19405022

[B3] Ashtiani M. Salehzadeh-Yazdi A. Razaghi-Moghadam Z. Hennig H. Wolkenhauer O. Mirzaie M. (2018). A systematic survey of centrality measures for protein-protein interaction networks. *BMC Syst. Biol.* 12:80. 10.1186/s12918-018-0598-2 30064421 PMC6069823

[B4] Banerjee-Basu S. Packer A. (2010). SFARI gene: an evolving database for the autism research community. *Dis. Model Mech.* 3 133–135. 10.1242/dmm.005439 20212079

[B5] Barabási A. L. (2007). Network medicine – from obesity to the “Diseasome”. *New Engl. J. Med.* 357 404–407. 10.1056/NEJMe078114 17652657

[B6] Bradshaw N. J. Hayashi M. A. (2017). NDE1 and NDEL1 from genes to (mal)functions: parallel but distinct roles impacting on neurodevelopmental disorders and psychiatric illness. *Cell. Mol. Life Sci.* 74 1191–1210. 10.1007/s00018-016-2395-7 27742926 PMC11107680

[B7] Choobdar S. Ahsen M. E. Crawford J. Tomasoni M. Fang T. Lamparter D. (2019). Assessment of network module identification across complex diseases. *Nat. Methods* 16 843–852. 10.1038/s41592-019-0509-5 31471613 PMC6719725

[B8] Fabregat A. Sidiropoulos K. Viteri G. Forner O. Marin-Garcia P. Arnau V. (2017). Reactome pathway analysis: a high-performance in-memory approach. *BMC Bioinformatics* 18:142. 10.1186/s12859-017-1559-2 28249561 PMC5333408

[B9] Fasano M. Monti C. Alberio T. (2016). A systems biology-led insight into the role of the proteome in neurodegenerative diseases. *Expert Rev. Proteomics* 13 845–855. 10.1080/14789450.2016.1219254 27477319

[B10] Kleinberg J. M. (1999). Authoritative sources in a hyperlinked environment. *J. ACM* 46 604–632. 10.1145/324133.324140

[B11] Labonne J. Driessen T. M. Harris M. E. Kong I. K. Brakta S. Theisen J. (2020). Comparative genomic mapping Implicates LRRK2 for intellectual disability and autism at 12q12, and HDHD1, as well as PNPLA4, for X-Linked intellectual disability at Xp22.31. *J. Clin. Med.* 9:274. 10.3390/jcm9010274 31963867 PMC7019335

[B12] Liao Y. Wang J. Jaehnig E. J. Shi Z. Zhang B. (2019). WebGestalt 2019: gene set analysis toolkit with revamped UIs and APIs. *Nucleic Acids Res.* 47 W199–W205. 10.1093/nar/gkz401 31114916 PMC6602449

[B13] Ma J. Shojaie A. Michailidis G. (2019). A comparative study of topology-based pathway enrichment analysis methods. *BMC Bioinformatics* 20:546. 10.1186/s12859-019-3146-1 31684881 PMC6829999

[B14] Monti C. Colugnat I. Lopiano L. Chiò A. Alberio T. (2018). Network analysis identifies disease-specific pathways for Parkinson’s disease. *Mol. Neurobiol.* 55 370–381. 10.1007/s12035-016-0326-0 28004338

[B15] Monti C. Zilocchi M. Colugnat I. Alberio T. (2019). Proteomics turns functional. *J. Proteomics* 198 36–44. 10.1016/j.jprot.2018.12.012 30553948

[B16] Piñero J. Berenstein A. Gonzalez-Perez A. Chernomoretz A. Furlong L. I. (2016). Uncovering disease mechanisms through network biology in the era of next generation sequencing. *Sci. Rep.* 6:24570. 10.1038/srep24570 27080396 PMC4832203

[B17] Pramparo T. Lombardo M. V. Campbell K. Barnes C. C. Marinero S. Solso S. (2015). Cell cycle networks link gene expression dysregulation, mutation, and brain maldevelopment in autistic toddlers. *Mol. Syst. Biol.* 11:841. 10.15252/msb.20156108 26668231 PMC4704485

[B18] R Core Team (2017). *R: A Language and Environment for Statistical Computing.* Vienna: R Foundation for Statistical Computing.

[B19] Sharma A. Cinti C. Capobianco E. (2017). Multitype network-guided target controllability in phenotypically characterized osteosarcoma: role of tumor microenvironment. *Front. Immunol.* 8:918. 10.3389/fimmu.2017.00918 28824643 PMC5536125

[B20] Sonawane A. R. Weiss S. T. Glass K. Sharma A. (2019). Network medicine in the age of biomedical big data. *Front. Genet.* 10:294. 10.3389/fgene.2019.00294 31031797 PMC6470635

[B21] Su G. Morris J. H. Demchak B. Bader G. D. (2014). Biological network exploration with Cytoscape 3. *Curr. Protoc. Bioinformatics* 47 8.13.1–8.13.24. 10.1002/0471250953.bi0813s47 25199793 PMC4174321

[B22] Subramanian A. Tamayo P. Mootha V. K. Mukherjee S. Ebert B. L. Gillette M. A. (2005). Gene set enrichment analysis: a knowledge-based approach for interpreting genome-wide expression profiles. Version 2. *Proc. Natl. Acad. Sci. U.S.A.* 102 15545–15550. 10.1073/pnas.0506580102 16199517 PMC1239896

[B23] Suwinski P. Ong C. K. Ling M. H. T. Poh Y. M. Khan A. M. Ong H. S. (2019). Advancing personalized medicine through the application of whole exome sequencing and big data analytics. *Front. Genet.* 10:49. 10.3389/fgene.2019.00049 30809243 PMC6379253

[B24] Tebani A. Afonso C. Marret S. Bekri S. (2016). Omics-based strategies in precision medicine: toward a paradigm shift in inborn errors of metabolism investigations. *Int. J. Mol. Sci.* 17:1555. 10.3390/ijms17091555 27649151 PMC5037827

[B25] Wang X. Liang S. Fujisawa T. X. Nishitani S. Tomoda A. Zou M. (2016). Association of estrogen receptor alpha polymorphisms with symptoms of autism among Chinese Han children. *Neuro Endocrinol. Lett.* 37 439–444. 28315628

[B26] Yu G. He Q.-Y. (2016). ReactomePA: an R/Bioconductor package for reactome pathway analysis and visualization. *Mol. Biosyst.* 12 477–479. 10.1039/c5mb00663e 26661513

