# Supplementary figures and images for: Gene Set Enrichment Analysis of Interaction Networks Weighted by Node Centrality
Source: Front Genet. 2021 Feb 24;12:577623. doi: 10.3389/fgene.2021.577623 (PMC7943873; doi:10.3389/fgene.2021.577623)

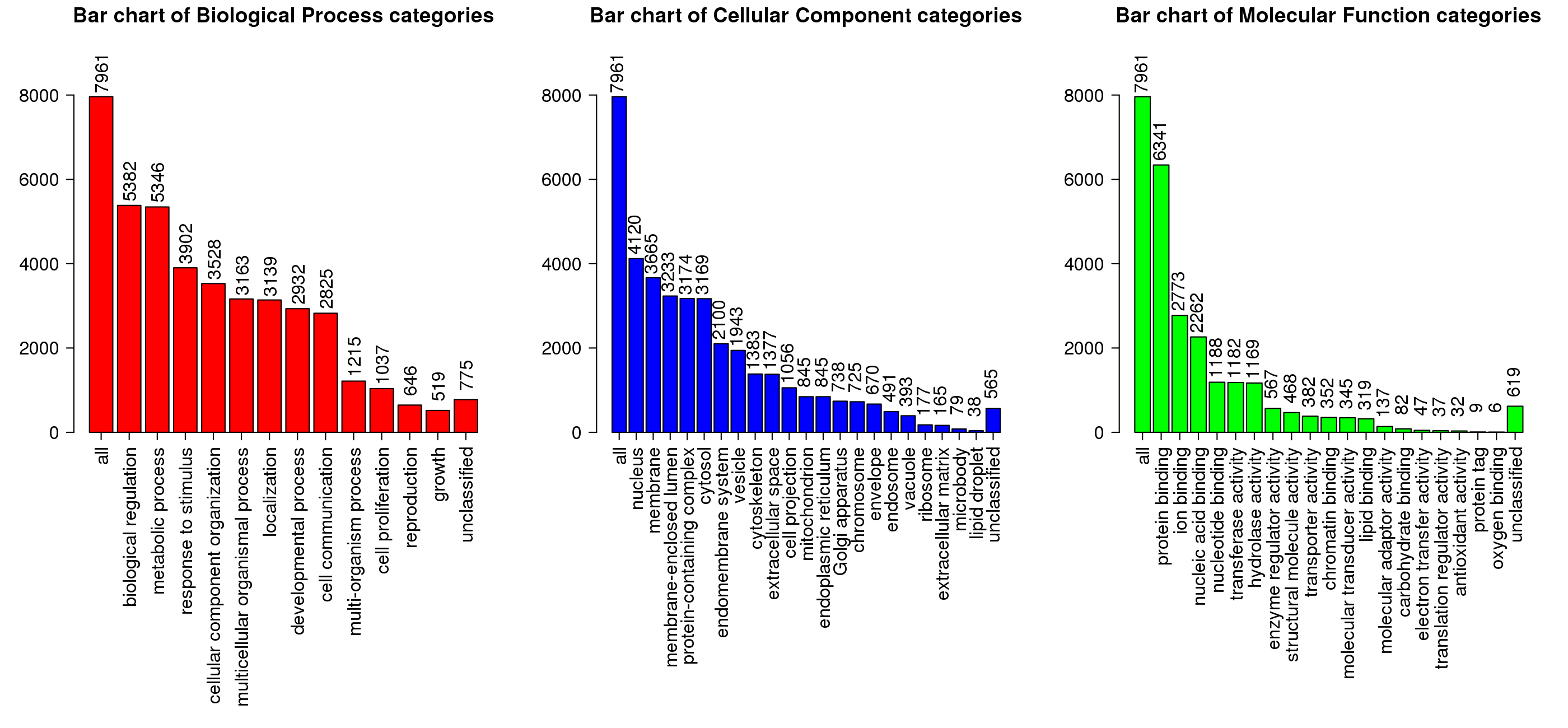

Supplement: Supplementary file 1 [file Data_Sheet_1.ZIP › goslim_summary_wg_result1608311829.png]

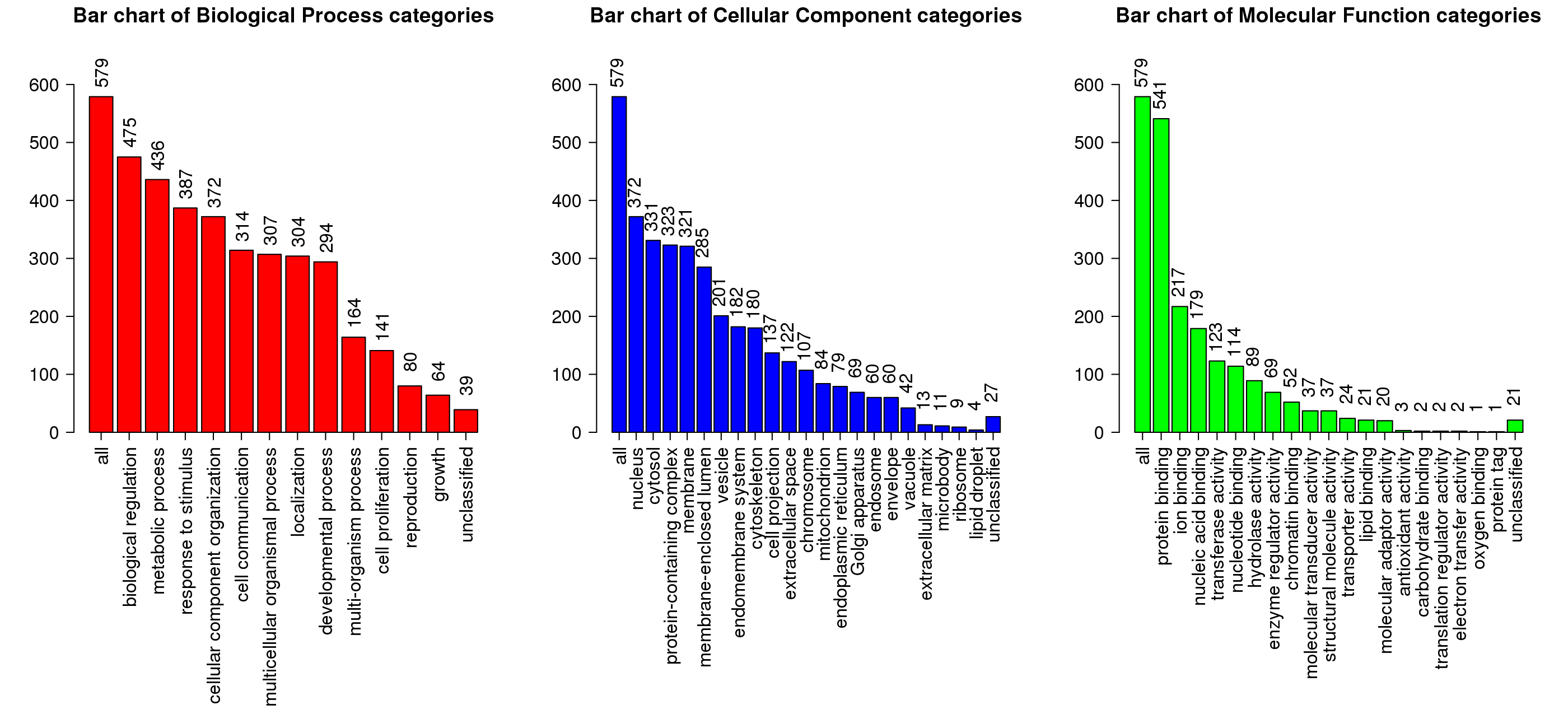

Supplement: Supplementary file 2 [file Data_Sheet_2.ZIP › goslim_summary_wg_result1608310350.png]

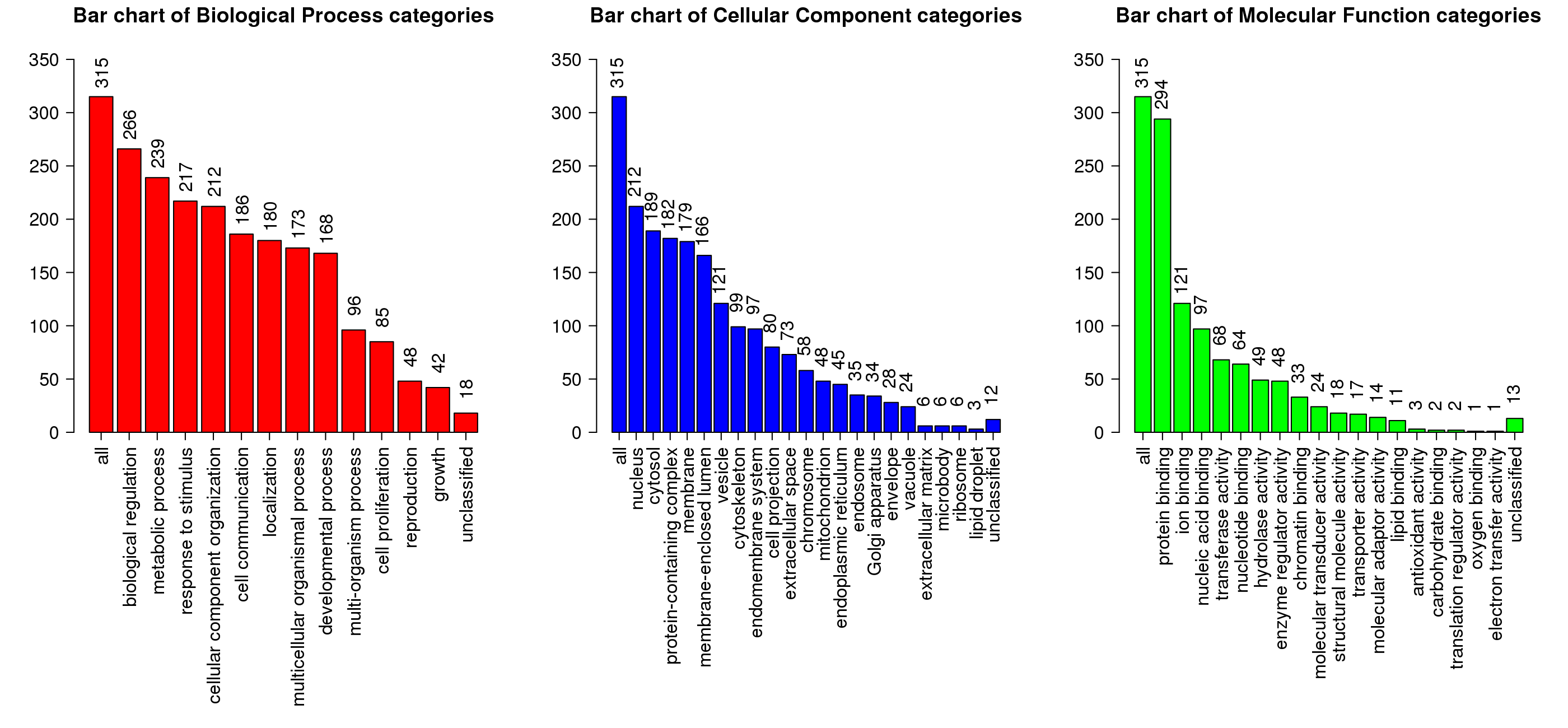

Supplement: Supplementary file 3 [file Presentation_3.zip › goslim_summary_wg_result1608310649.png]

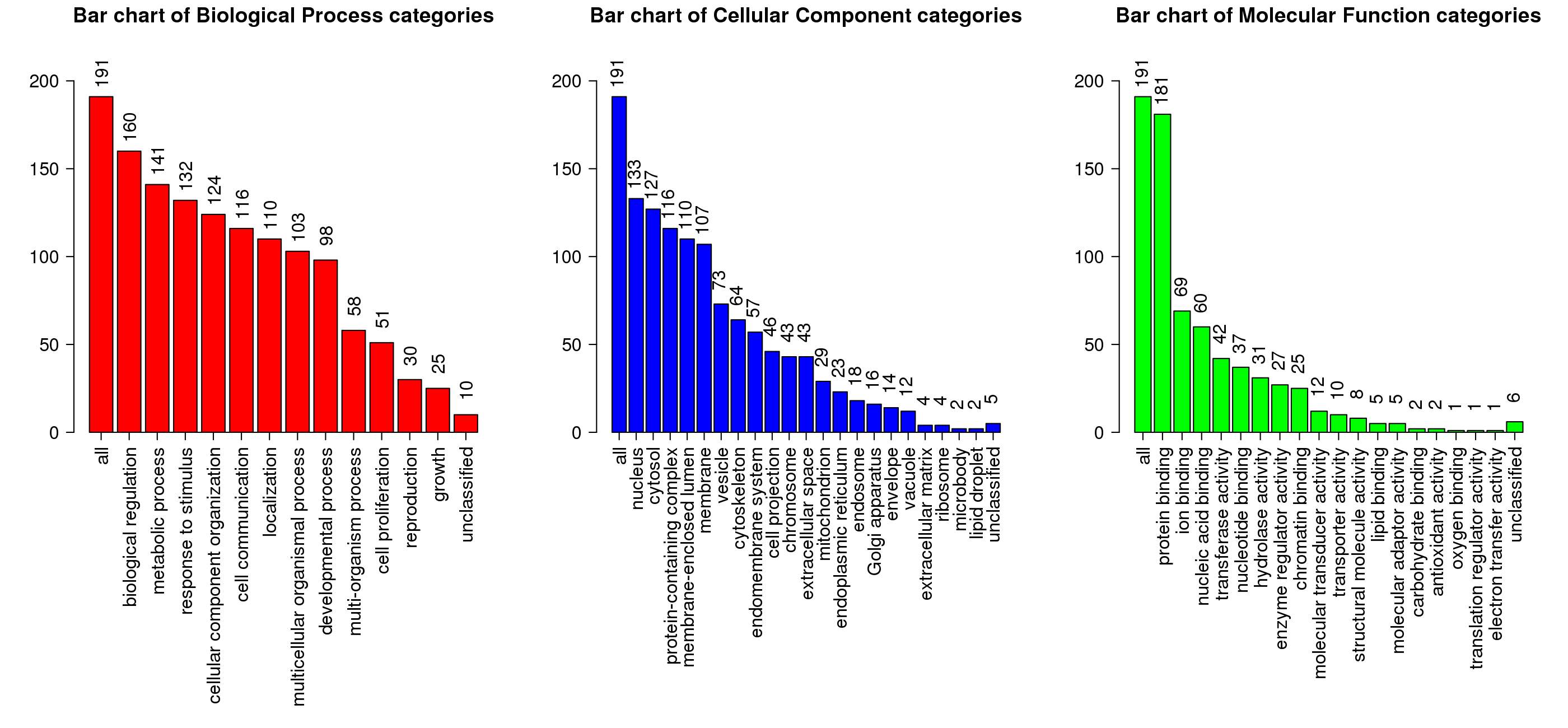

Supplement: Supplementary file 4 [file Presentation_4.zip › goslim_summary_wg_result1608310845.png]
