# Supplementary material for: Gene Set Enrichment Analysis of Interaction Networks Weighted by Node Centrality
Source: Front Genet. 2021 Feb 24;12:577623. doi: 10.3389/fgene.2021.577623 (PMC7943873; doi:10.3389/fgene.2021.577623)
Supplement: Supplementary file 5 [file Data_Sheet_5.docx]

**Gene Set Enrichment Analysis of interaction networks weighted by node centrality**

**Supplementary Materials**

***Alessandra Zito,^1^ Marta Lualdi,^1^ Paola Granata,^2^ Dario Cocciadiferro,^3^ Antonio Novelli,^3^ Tiziana Alberio,^1^ Rosario Casalone,^2^ Mauro Fasano^1*^***

*^1^ Department of Science and High Technology, Center of Bioinformatics, University of Insubria, Busto Arsizio, Italy.*

*^2^ Unit of Cytogenetics and Medical Genetics, ASST dei Sette Laghi, Varese, Italy.*

*^3^ Laboratory of Medical Genetics, Ospedale Pediatrico Bambino Gesù, Rome, Italy.*

^*^ Correspondence: Mauro Fasano. mauro.fasano@uninsubria.it

Keywords: systems medicine; network medicine; gene set enrichment analysis; topological analysis; protein-protein interaction.

**R code**

# example: file PD_node_all.csv contains centrality values of the network

# in figure 4C. Exported from Cytoscape.

rm(list=ls())

setwd("~home/database")

database<-read.csv(file="~/data/HS_genome.csv", sep=";", header=T)

subnet<-"PD"

mydata<-read.csv(file=paste(subnet, "_node_all.csv", sep=""), sep=",")

# remove entries that cannot be translated

if (length(grep("EBI", mydata$name))>0)

{mydata<-mydata[-grep("EBI", mydata$name),]}

if (length(grep("hCG", mydata$name))>0)

{mydata<-mydata[-grep("hCG", mydata$name),]}

if (length(grep("Em:", mydata$name))>0)

{mydata<-mydata[-grep("Em:", mydata$name),]}

desc1<-as.character(mydata$name)

centr1<-as.numeric(mydata$BetweennessCentrality)

# translate IDs into geneIDs

g<-length(desc1)

library(rentrez)

symb1<-as.character()

for (i in 1:g) {

if (length(which(database$Symbol==desc1[i]))>0)

{symb1[i]<-database$GeneID[which(database$Symbol==desc1[i])][1]}

else

{

if (length(grep("ENS",desc1[i]))==1)

{try(symb1[i]<-entrez_search("gene",desc1[i])$ids)}

else

{

A<-grep(desc1[i],database$Aliases, value=T)

B<-strsplit(A, ", ")

C<-integer()

if (length(B)>0) {

for (k in 1:length(B))

{C[k]<-length(which(unlist(B[[k]])==desc1[i]))}

D<-grep(desc1[i],database$Aliases)[which(C==1)]

try(symb1[i]<-database$GeneID[D])

}

else {symb1[i]<-NA}

}

}

if(i %% 100 ==T)

{ print(i)}

}

miss<-which(is.na(symb1))

library(org.Hs.eg.db)

mylist<-select(org.Hs.eg.db, keys=desc1[miss], columns="ENTREZID", keytype="SYMBOL")

symb1[miss]<-mylist$ENTREZID

# build a named vector of centrality values

desc2<-desc1[order(-centr1)]

geneList<-centr1[order(-centr1)]

geneid<-symb1[order(-centr1)]

names(geneList)<-geneid

# Hill transformation (h=1.6, k=1E-3)

h<-1.6

k<-1e-3

gl<-(geneList^h)/(k+geneList^h)

gl<-(gl-min(gl))/(max(gl)-min(gl))

names(gl)<-geneid

extr<-cbind(desc2, geneid, as.numeric(geneList), as.numeric(gl))

write.csv(extr, file=paste(subnet,"centr_s_new.csv", sep=""))

library(DOSE)

library(ReactomePA)

library(stats)

# perform GSEA

y <- gsePathway(gl, pvalueCutoff=0.5, pAdjustMethod="BH", verbose=FALSE)

# dot plot

tiff(paste(patient,"_DOT_path.tif", sep=""),height = 1200, width = 1200)

dotplot(y, color="pvalue")

dev.off()

# export results as a table

gsea_results<-y@result

write.csv(gsea_results, file=paste(subnet,"_gsea_tot_s.csv", sep=""))

# Resampling

randompos<-sample(1:g, g, replace=F)

glR<-gl[randompos]

names(glR)<-geneid

glR<-glR[order(-glR)]

# GSEA on resampled gene list

yR <- gsePathway(glR, pvalueCutoff=1, pAdjustMethod="BH", verbose=FALSE)

tiff(paste(subnet,"_RANDOM_h_",h,"_k_",k,"_DOT_path.tif", sep=""),height = 1200, width = 1200)

dotplot(yR, color="pvalue", showCategory=10, font.size=24)

dev.off()


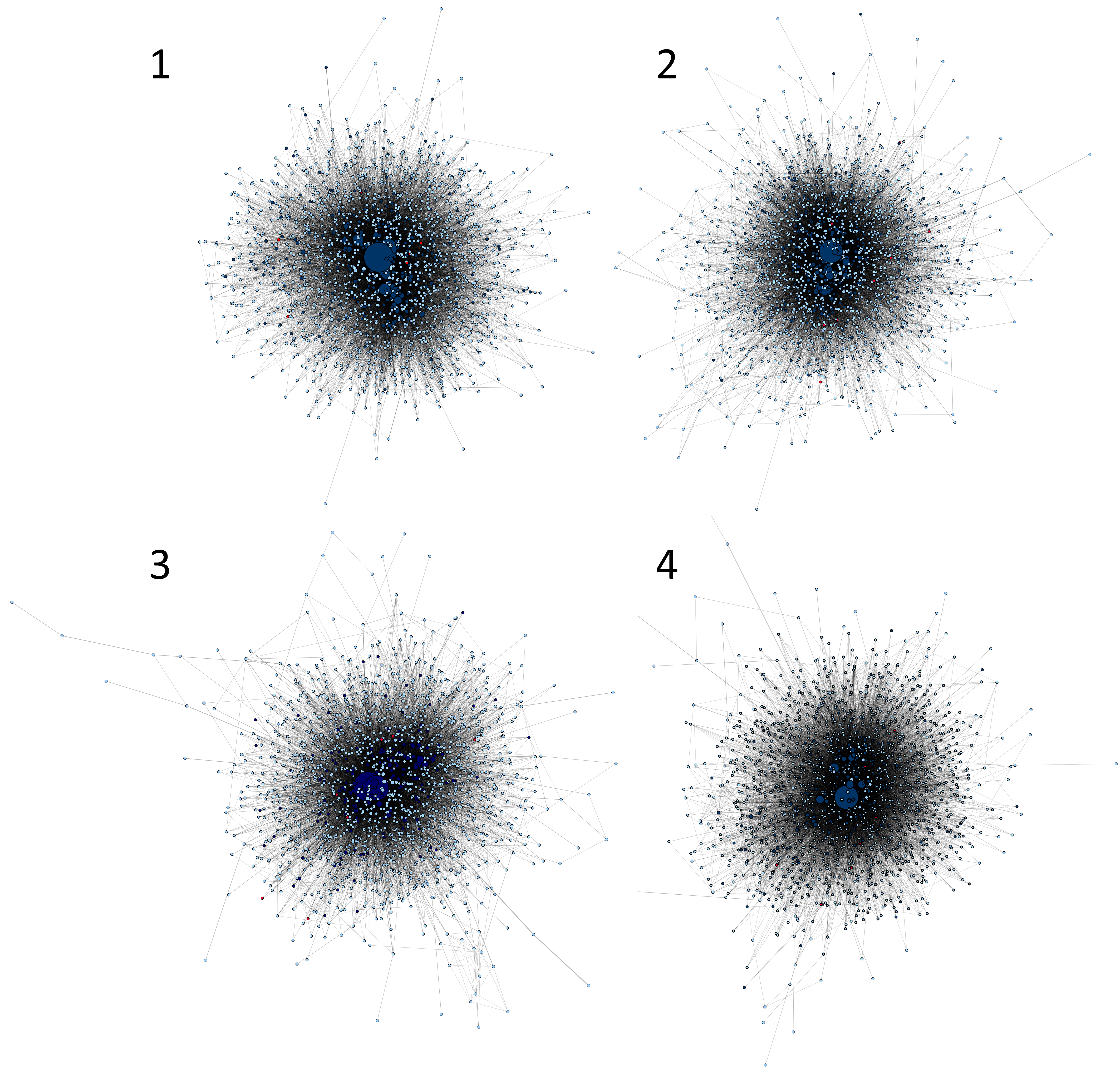


**Supplementary Figure 1:** Personalized patients’ networks of altered genes and their interactors. Numbers refer to patients as indicated in Table 1. Terminal (degree = 1) and isolated (degree = 0) nodes were removed before topological analysis except for signature nodes. Red nodes are genes from the individual gene signature, navy blue nodes are their first interactors and light blue nodes are second interactors. Diameter and border width of nodes are proportional to betweenness centrality, whereas edge width is proportional to edge betweenness. For clarity reasons, only nodes with *C_B_* > 10^-4^ are shown.





**Supplementary Figure 2:** Centrality metrics before and after Hill transformation. The solid line shows closeness centrality obtained from the topological analysis of a representative personalized network. The dashed line represents centrality values after transformation with a Hill function.


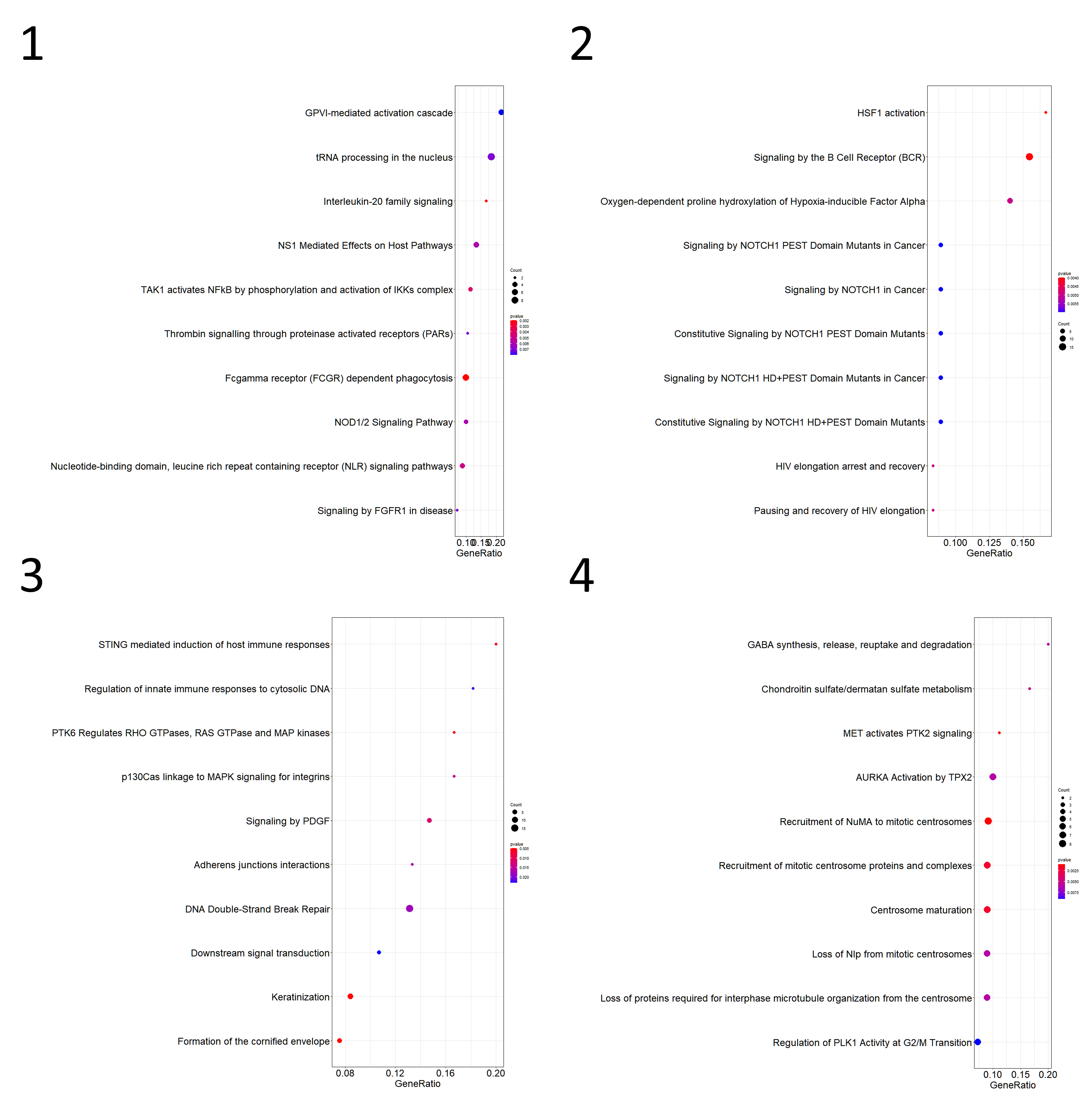


**Supplementary Figure 3:** Dot plots for the four GSEA results after resampling. Numbers refer to patients as indicated in Table 1. Dot size is proportional to the number of overlapping genes. p-values are color-coded according to the color scale.


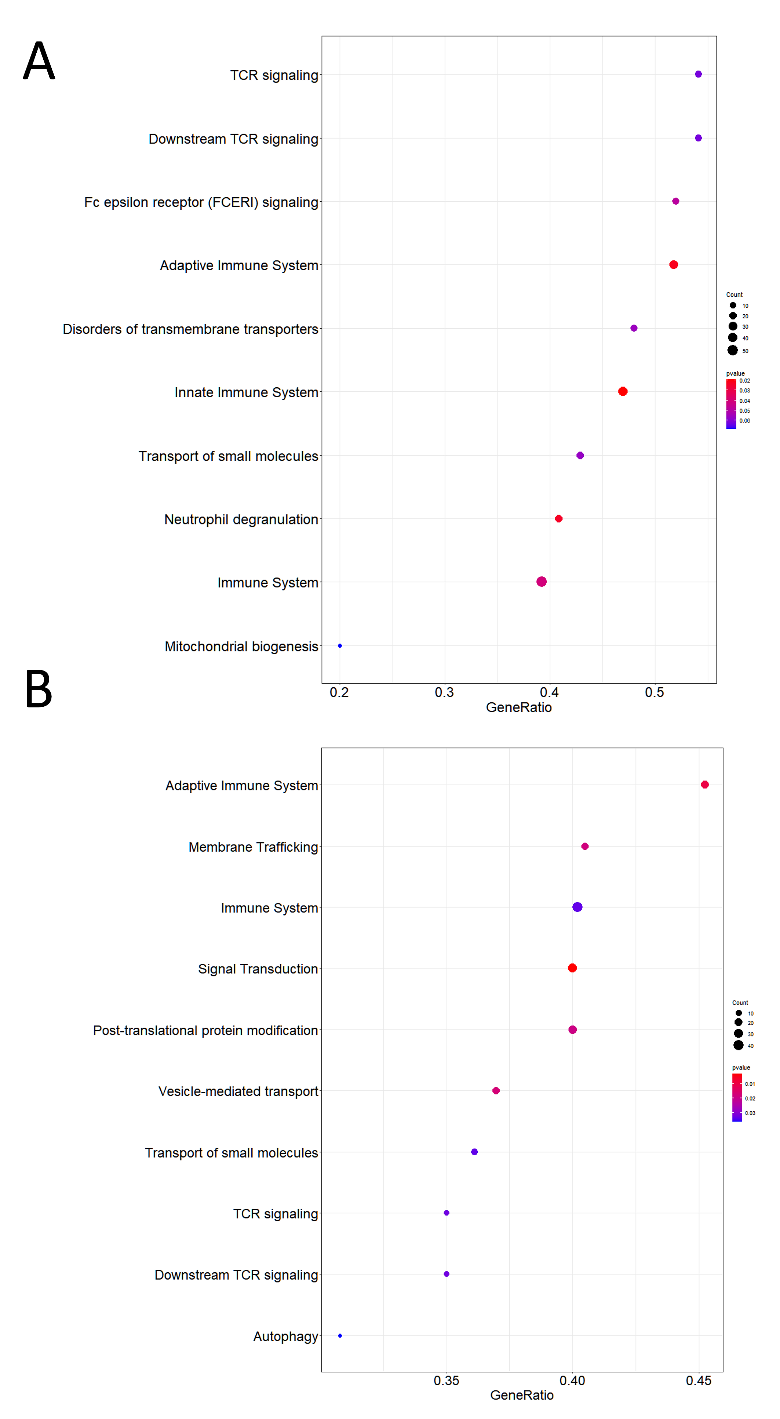


**Supplementary Figure 4.** Dot plots for the GSEA results after resampling. A: whole network; B: selection of nodes altered in Parkinson’s disease patients. Dot size is proportional to the number of overlapping genes. P-values are color-coded according to the color scale.
